# Risk of carditis among adolescents after extending the interdose intervals of BNT162b2

**DOI:** 10.1038/s41541-023-00789-6

**Published:** 2024-02-14

**Authors:** Min Fan, Kuan Peng, Yin Zhang, Francisco Tsz Tsun Lai, Celine Sze Ling Chui, Eric Yuk Fai Wan, Carlos King Ho Wong, Esther Wai Yin Chan, Xue Li, Ian Chi Kei Wong

**Affiliations:** 1 Centre for Safe Medication Practice and Research, Department of Pharmacology and Pharmacy, Li Ka Shing Faculty of Medicine, The University of Hong Kong, Pok Fu Lam, Hong Kong China; 2 Department of Medicine, School of Clinical Medicine, Li Ka Shing Faculty of Medicine, University of Hong Kong, Pok Fu Lam, Hong Kong China; 3 Laboratory of Data Discovery for Health (D24H), Hong Kong Science Park, Pok Fu Lam, Hong Kong China; 4 School of Nursing, Li Ka Shing Faculty of Medicine, University of Hong Kong, Pok Fu Lam, Hong Kong China; 5 School of Public Health, Li Ka Shing Faculty of Medicine, University of Hong Kong, Pok Fu Lam, Hong Kong China; 6 Department of Family Medicine and Primary Care, School of Clinical Medicine, Li Ka Shing Faculty of Medicine, University of Hong Kong, Pok Fu Lam, Hong Kong China; 7 Research Department of Practice and Policy, UCL School of Pharmacy, London, UK; 8 Aston Pharmacy School, Aston University, Birmingham, UK

**Keywords:** Drug development, Cardiovascular diseases

## Abstract

Previous studies indicate an increased carditis risk among adolescents following the two-dose messenger RNA COVID-19 vaccine. Several jurisdictions have extended the interdose interval between the first and second doses to reduce the risk. However, the effectiveness of such an extension policy remains inconclusive. Using the territory-wide vaccine record-linked electronic health records in Hong Kong, we conducted a nested case–control study from February 23, 2021 to August 15, 2022. Adolescents aged between 12 and 17 who received two-dose BNT162b2 were included for comparing risks between standard interdose interval (21–27 days) versus extended interdose interval ( ≥ 56 days). The carditis cumulative incidence within 28 days following the second dose was calculated. The adjusted odds ratio was estimated from multivariable conditional logistic regression. We identified 49 adolescents with newly diagnosed carditis within 28 days following the second dose. The crude cumulative incidence is 37.41 [95% confidence interval (CI): 27.68–49.46] per million vaccinated adolescents. Compared to the standard interdose interval group, adolescents with an extended interval had a significantly lower risk of carditis [adjusted odds ratio (aOR) 0.34 (95% CI: 0.16–0.73)]. Sensitivity analysis of carditis occurring within 14 days following the second dose yielded a similar estimate [aOR 0.30 (95% CI: 0.13–0.73)]. Extending the interdose interval of the BNT162b2 vaccine from 21 to 27 days to 56 days or longer is associated with 66% lower risk of incident carditis among adolescents. Our findings contribute towards an evidence-based vaccination strategy for a vulnerable population and potentially informs product label updates.

Both case reports and comparative studies have reported an increased risk of carditis following the second dose of mRNA-1273 and BNT162b2, the messenger RNA (mRNA) COVID-19 vaccine^1,2^, particularly among male adolescents^3–15^. Possible solutions for this unfavorable vaccine-associated adverse event could be the use of a single dose only or extending the interdose interval. Our previous work suggested the effectiveness of a single-dose policy in reducing the risk of carditis before the Omicron circulation^16^. Given that the pandemic is shifting towards an endemic stage and thus, a continued need for routine COVID-19 vaccination, extending the interdose interval may be a better option according to some experts^1,17^. However, current evidence about dose interval is graded as low certainty^1^. To date, there is only one descriptive cohort study in Canada reporting a lower crude rate of carditis among adolescents when the interval becomes 56 or above, compared with 30 or fewer days (9.6 vs 52.1 cases per 1,000,000 doses)^18^.

In Hong Kong, BNT162b2 was first made available to adolescents in June 2021 with a recommended dose interval of 21 days, the same as for adults^19^. However, a local pharmacovigilance study revealed an increased risk of incident carditis among two-dose vaccine recipients, particularly in adolescents^8^. To maintain the benefits of vaccination during the pandemic while minimizing potential adverse reactions, the Department of Health extended the recommended dose interval to 56 days from March 2022^20^. There is neither existence of a universal standard on the vaccination interval (range from 21 days to 84 days across the jurisdictions in Supplementary Table 1), nor any real-world evidence could further support the impact of such an extension on vaccine-related carditis. Therefore, we conducted a nested case–control study using the territory-wide vaccine record-linked electronic health records in Hong Kong, aiming to assess the impact of an extending interdose interval on the risk reduction of vaccine-related carditis among adolescents.

Up to August 15, 2022, a total of 334,667 doses of BNT162b2 were administered to adolescents since the launch of the COVID-19 vaccination program in Hong Kong. Among 143,636 adolescents who received at least one dose of BNT162b2, 91.2% (*n* = 130,970) completed the second dose (42.7% with extended interdose intervals). Within the study period, 84 adolescents were hospitalized with carditis-related conditions. Following the exclusion criteria, we identified 49 incident carditis-related hospitalizations as the “cases”. The crude cumulative incidence of carditis within 28 days following the second dose was 37.41 (95% CI: 27.68–49.46) per 1,000,000 vaccinated adolescents. The 28-day cumulative incidence was higher in males than in female adolescents (57.13 vs 13.51 per 1,000,000 vaccinated adolescents, *P* = 0.013), lower in the extended interdose interval group than a standard interval group (19.65 vs 53.41 per 1,000,000 vaccinated adolescents, *P* = 0.034). In the subgroup analysis among male adolescents, the crude cumulative incidence of carditis was significantly lower in the extended interval group (22.44 vs 88.24 per 1,000,000 vaccinated adolescents, *P* < 0.001). In contrast, female adolescents with standard and extended intervals had similar cumulative incidences of carditis (12.27 vs 16.15 per 1,000,000 vaccinated adolescents, *P* = 0.733). The incident plot and detailed values are presented in Fig. 1 and Supplementary Table 2.

**Fig. 1:**
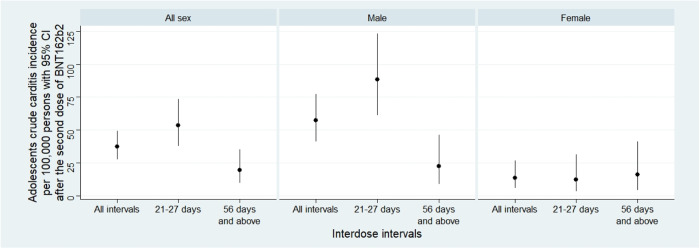
Cumulative incidence of carditis among adolescents following the second dose of BNT162b2. This is a plot for the adolescents crude carditis incidence per 100,000 persons with 95% confidence interval, group by sex. The circular data point represents the point estimation, while the error bar denotes the 95% confidence interval.

Figure 2 shows the selection flow of the nested case–control analysis. After matching, we identified 49 cases and 487 controls. The demographic information of study participants in the nested case–control is presented in Table 1. The majority of the participants were males (84.3% of control and 83.7% of cases), 79.6% of cases (*n* = 39) had myocarditis. Cases and controls had a similar percentage of patients with CVD or infection history; they also showed similar HA healthcare resource utilization patterns. Results from multivariable conditional logistic regression are presented in Table 2. Adolescents with the extended interdose interval had approximately 66% lower risk of carditis [aOR: 0.34 (0.16–0.73)]. In the two sensitivity analyses, a similar estimation was observed after changing the onset time to 14 days [aOR: 0.30 (0.13–0.73)] and removing cases and controls with an extreme interdose interval [aOR: 0.34 (0.15–0.77)] (Table 2). In the subgroup analysis by age, a lower risk [aOR: 0.20 (0.07–0.57)] was observed among adolescents aged 12 to 15 (Table 2).

**Fig. 2:**
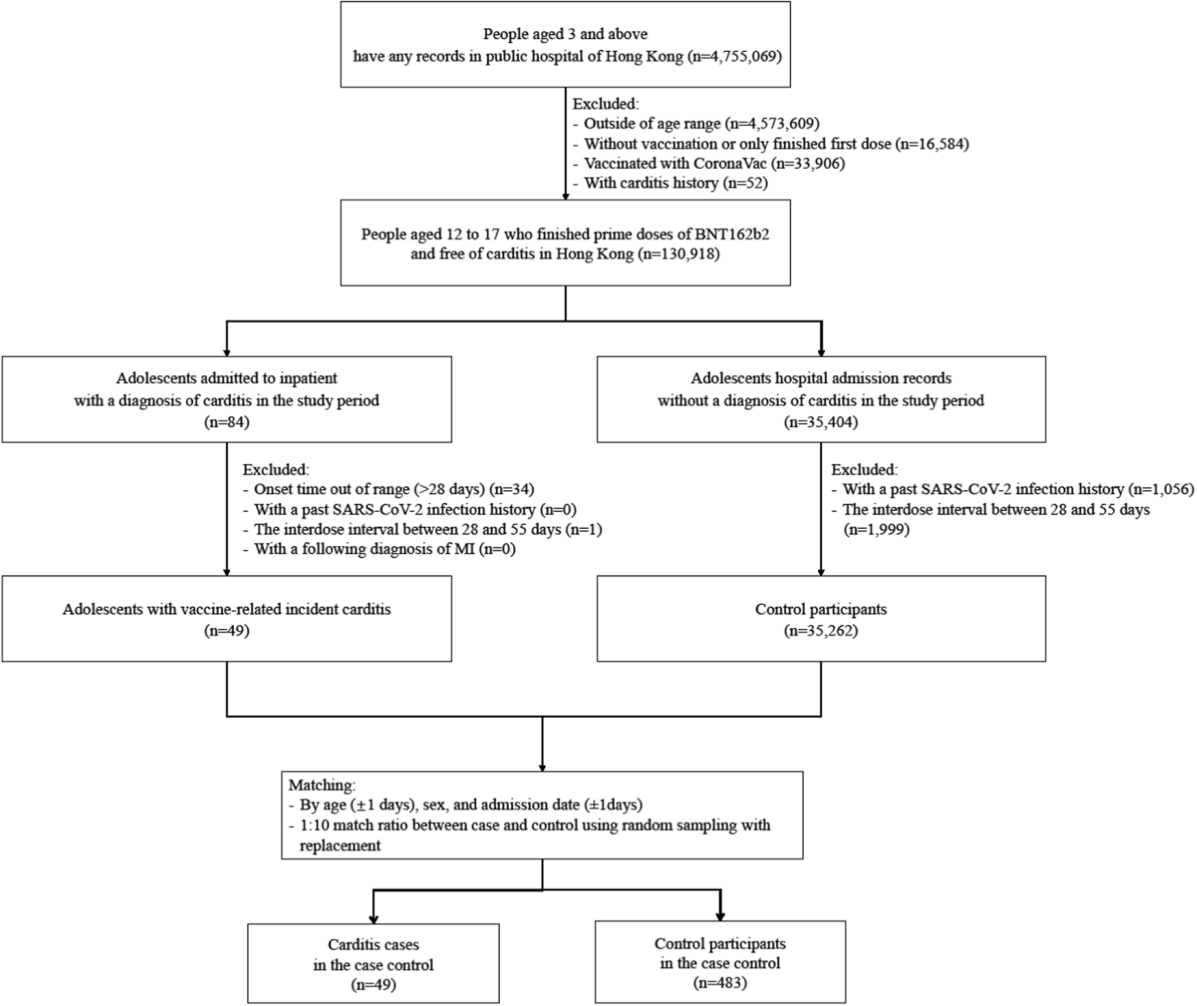
Identification of cases and controls. The flowchart of patient inclusion.

**Table 1:** Demographic and clinical characteristics of participants in the case–control analysis.

Characteristics	Controls	Cases	SMD*
	(*N* = 483)	(*N* = 49)	
Age, years [mean (SD)]	15.75 (1.46)	15.88 (1.52)	0.086
Sex, male (%)	407 (84.3)	41 (83.7)	0.016
Cardiovascular disease (%)	33 (6.8)	0 (0.0)	0.383
Past cardiovascular disease history			
Vascular disease	14 (2.9)	0 (0.0)	0.244
Myocardial infarction	0 (0.0)	0 (0.0)	–
Arrhythmia	2 (0.4)	0 (0.0)	0.091
Heart failure	0 (0.0)	0 (0.0)	–
Peripheral vascular disease	0 (0.0)	0 (0.0)	–
Coronary artery disease	0 (0.0)	0 (0.0)	–
Cardiovascular treatment within 180 days			
Statin	0 (0.0)	0 (0.0)	–
Angiotensin-converting enzyme	15 (3.1)	0 (0.0)	0.253
Angiotensin receptor blockers	2 (0.4)	0 (0.0)	0.091
Digoxin	0 (0.0)	0 (0.0)	–
Diuretics	0 (0.0)	0 (0.0)	–
Anticoagulant	0 (0.0)	0 (0.0)	–
Antiplatelet medications	0 (0.0)	0 (0.0)	–
Beta-blocker	0 (0.0)	0 (0.0)	–
Calcium channel blockers	0 (0.0)	0 (0.0)	–
Infections within 28 days before the index date	15 (3.1)	0 (0.0)	0.253
Healthcare resource utilization within 180 days [mean (SD)]			
Hospital admission	3.75 (11.64)	0.10 (0.31)	0.443
Outpatient consultation	2.16 (2.74)	0.31 (0.82)	0.917
A&E admission	0.62 (1.24)	0.35 (0.66)	0.276

*SMD standard mean difference, *A&E* Accidence and Emergency Department, *SD* standard deviation.

**Table 2:** Odds ratios of extended interdose interval for carditis risk from multivariable conditional logistic regressions.

Analysis	Unadjusted OR	aOR (95% CI)*
Primary analysis	0.31 (0.15–0.62)	0.34 (0.16–0.73)
Sensitivity analysis		
Onset time from 28 days to 14 days	0.23 (0.10–0.52)	0.30 (0.13–0.73)
Removing extreme interdose interval	0.36 (0.17–0.74)	0.34 (0.15–0.77)
Subgroup analysis		
Age between 12 and 15 (*n* = 341)	0.19 (0.07–0.51)	0.20 (0.07–0.57)
Age between 16 and 18 (*n* = 191)	0.60 (0.22–1.64)	0.70 (0.19–2.58)

*OR odds ratio, *aOR* adjusted odds ratio.

*The estimation was adjusted by cardiovascular disease history, pre-infection within 28 days, outpatient records number, accident and emergency admission records number, and inpatient records within 180 days.

To the best of our knowledge, this is the first analytic study assessing the risk of carditis among adolescents receiving priming doses BNT162b2 at different interdose intervals. We observed a significantly lower cumulative incidence of carditis among adolescents who received the second dose with an extended interval (56 days or above) compared with those who had undergone the standard interval (21–27 days). This was further confirmed in the nested case–control analysis, from which the risk reduction associated with extended dosing interval was estimated to be 66%.

Our findings suggest young males are at higher risk of mRNA vaccine-related carditis although the absolute risk is low, which aligns with similar studies conducted worldwide^3,4,8,18,21,22^. In the USA, the 7-day risk of myocarditis following the second dose of BNT162b2 is higher among adolescents than adults. The rate is much higher for males than female adolescents [70.73 (6.16–81.11) vs 6.35 (4.05–9.96) per million doses in the 12–15 age group]^3^. Our previous study also reported that compared with unvaccinated individuals, the odds of carditis following BNT162b2 were greater among adolescents [aOR: 13.79 (2.86–110.38)] than that for adults (aOR: 2.41, 1.18–4.90)^8^. The mechanism of mRNA vaccine-related carditis is under investigation. A possible mechanism is the cross-reaction between myocardial α-myosin heavy chain and antibodies directed to the spike protein of SARS-CoV-2 from the mRNA vaccines^4,21^. Another recent study with endomyocardial biopsy from Germany proposed that endogenous interleukin-1 receptor antagonists (IL-1RA) may be one of the triggers of carditis. Anti-IL-1RA antibodies were found in 75% (9 of 12) of samples from younger individuals, which is consistent with the higher cumulative incidence among adolescents^23^. Our subgroup analysis shows that the effect is stronger in the younger adolescents between 12 and 15. However, the lack of significance in the older age group may be due to a smaller sample size, and further studies is warranted. In addition, recent findings from Connectivity Map indicate that BNT162b2 may impact calcium homeostasis, elevating carditis risk. In addition, interactions with sex steroid hormones could explain sex-specific differences in susceptibility^23,24^.

The reported incidence of mRNA vaccine-related carditis among adolescents ranged from 9.0 per million doses administered in the UK^22^, to 53.6 in Canada and 105.9 in the USA^3,18^. These considerable variations could be explained by the difference in outcome definition (myocarditis versus all carditis), onset time (7 versus 28 days), age cutoffs (12–17 versus 16–17 years), and notably, the correlation with recommended interdose interval of priming doses (8 weeks in the UK versus 3 or 4 weeks in Canada and the USA)^25–27^. Realizing the risk of carditis following mRNA COVID-19 vaccines, an increasing number of countries are considering extending the interdose intervals to reduce the risk of adverse reactions^28–30^. However, at the time of study, there was no universal standard (Supplementary Table 1) or planned product label updates for the optimized dosing intervals. The impact of extended dosing intervals needs to be carefully evaluated and disseminated. To date, only one descriptive study reported a reduced crude incidence of carditis following the extended interdose interval among adolescents in Canada. The overall carditis rate after BNT162b2 is higher when the interval was 30 days or fewer [52.1 (31.8–80.5) cases per million doses], compared with 56 days or more [9.6 (6.5–13.6) cases per million doses]^18^. The Canadian study’s provided early empirical evidence that is important for interdose consideration; however, the authors also pointed out the limitations inherent to passive vaccine-safety surveillance systems such as augmented reporting rate^18^. Furthermore, the Canadian study did not control previous medical history and Covid infection. Using a population-based, territory-wide active safety surveillance database, our study confirmed the observation from Canada with individual-level clinical and demographical factors adjusted^18^. The estimated risk reduction associated with the extended interdose interval is 66% and robust with a stringent onset time of carditis (14 days since the second dose), which would provide reassurance of the effectiveness and need for an extended interdose interval in adolescents.

Currently, policies on interdose interval of the BNT162b2 vaccine for adolescents vary worldwide (Supplementary Table 1). Some countries recommend longer intervals of 8 weeks or more, including Canada, Australia, Singapore, Hong Kong, the UK, Norway, and Taiwan. However, some still recommend shorter intervals, such as Japan, Germany, and Finland. Although timely completion of immunization may prevent some additional cases in the short term, according to our study, a longer 56-day interdose interval could help reduce the risk of carditis in adolescents. Meanwhile, our another study has shown that extended dose interval is associated with 29.2% of risk reduction of Covid-19 infection in adolescents, possibly due to improved immunogenicity^31^. In addition, for resource-poor areas with limited vaccine supplies, extending the second dose allows for higher first dose coverage in response to urgent public health needs during outbreaks. Considering such a significant safety and effectiveness impact, we recommend adolescents take the first dose as soon as possible during the pandemic (in the foreseeable future) to obtain timely protection, and the second dose interval could be longer than the regularly recommended 28 days to maintain the risk-benefit balance. Further studies may consider evaluating the long-term safety of the extended interdose interval and extending studies to other sites to validate the benefits of the extended dose interval policy.

This study was also subject to several limitations. First, this study adopted a nested case–control design to measure the association with rare carditis events. Due to the small numbers of carditis, we do not have an adequate sample size to investigate further the effect of extending the interdose interval by sex and disease type (i.e., myocarditis or pericarditis). Second, the selection of controls for our study are hospitalized adolescents during the same study period. They were deemed to be less healthy than adolescents without hospitalization records, thus could introduce a selection bias. Third, although we have conducted multivariable adjustment for the nested case–control study, similar to all pharmacoepidemiological studies, we cannot exclude unmeasured confounders, such as social, economic status or health-seeking behaviors among adolescents^32^. Fourthly, this is an observational study utilizing electronic health records potentially suffered from unmeasured confounding factors such as socio-economic status, environmental and genetic factors that are not included in the database. Further analysis by other sites is warranted to validate these findings. Lastly, the ascertainment of carditis is based on ICD-9 diagnosis codes only that might result in misdiagnosis of carditis. Like most of electronic health records database, image, and clinical notes are not available in our dataset and we can only use ICD-9 for case identification. However, we would like to emphasize that the all the diagnosis was made by pediatric cardiologists and the diagnosis standards were especially stringent for cases of myocarditis and pericarditis, given their inclusion in the list of Adverse Events of Special Interests (AESIs) for COVID-19 and the safety labeling of BNT162b2 under emergency use^33^. The impact of using ICD-9 as the diagnosis criteria is expected to have minimal impact on the conclusions drawn in this paper.

## Methods

### Data source

We obtained clinical information on the diagnosis, prescription, laboratory results, emergency department attendance, and hospitalization details from the Hospital Authority (HA), a statutory body funding healthcare and acute care provider in Hong Kong. The HA provides publicly funded health services to over 7.4 million Hong Kong residents, with 43 public hospitals, 49 specialist outpatient clinics and 73 primary care clinics^34,35^. All Hong Kong residents are eligible to access publicly subsidized healthcare services provided by the HA. The current HA dataset covers all data in public hospitals up to August 15, 2022. The vaccination records were provided by the Department of Health, which oversees all COVID-19 mass vaccination programs in Hong Kong. The BNT162b2 vaccination program for adolescents began in June 2021^19,36^. The minimal age thresholds were extended to all residents ≥16 years on April 15, 2021 and all residents ≥12 years on June 11, 2021^19,37^. During the study period, the Hong Kong government took responsibility for the massive COVID-19 vaccination program within the region. Owing to the imposition of travel restrictions and quarantine policies, adolescents faced limited opportunities to receive vaccinations through alternative channels outside of this established system.A deidentified unique pseudo ID was used to match vaccination and health records. This dataset has been used to evaluate COVID-19 vaccine studies on carditis safety^8,11,16,38^.

### Study design

This is a population-based nested case–control study comparing the risk of carditis between standard (21–27 days) and extended (56 days and above) interdose intervals. The underlying cohort includes all Hong Kong adolescents, aged between 12 and 17 years old, who had used HA services and completed two doses of BNT162b2. We defined cases as the participant with a carditis diagnosis [International Classification of Diseases, Ninth Revision, Clinical Modification (ICD-9-DM): 422 and 429.0 for myocarditis, 420.9 and 423.9 for pericarditis] from the inpatient setting between February 23, 2021 and August 15, 2022. We excluded participants (1) with a past history of carditis to ensure only incident cases were analyzed; (2) with a carditis admission date more than 28 days since the second dose vaccination to ensure it is Covid-19-vaccine-related; (3) with a positive SARS-CoV-2 polymerase chain reaction test result before index date to eliminate the effects of a viral infection; (4) with an uncommon interdose interval not defined previously (28–55 days, inclusive); and (5) with a subsequent diagnosis of myocardial infarction (ICD-9-DM: 410 and 411) within the same hospitalization episode, as similarities in the presentation may cause misdiagnosis of carditis. The index date of cases was the admission date of carditis hospitalization. Within the underlying cohort, hospitalized adolescents without any carditis diagnosis during the study period were identified as controls. The exclusion criteria were the same as that for cases. Up to ten controls were randomly selected and matched with each case according to age (with one-year interval), date of admission (with one calendar day) and sex. The index date of controls was defined as their matched admission date. Incident density sampling was applied: cases were allowed to be controls before their incident carditis.

### Statistical analyses

We calculated the crude cumulative incidence of carditis within 28 days following the second dose of BNT162b2. The crude cumulative incidence was calculated by incident number divided by vaccinated adolescent counts, with subgroup analysis by sex. The comparison was conducted with the Fisher Exact test, and the 95% confidence interval was calculated based on the Poisson distribution.

In the nested case–control analyses, a carditis risk comparison was made between adolescents with standard and extended interdose intervals. Conditional logistic regression was used to estimate the odds ratio and 95% CI, with adjustment of cardiovascular disease (CVD) history, recent infection records and healthcare utilization. A binary CVD indicator was created if the adolescent had any CVD diagnostic history or had CVD-related prescription records 180 days before the index date. The recent infection records considered all the infection diagnosis records 28 days before the index date. The recent healthcare resource utilization included the number of accident and emergency admissions, the number of inpatient hospitalizations, and the number of outpatient visits in the past 180 days. The International Classification of Diseases, Ninth Revision, Clinical Modification (ICD-9-CM), and British National Formulary codes used to identify the history and prescription are presented in Supplementary Table 3.

In the sensitivity analyses, we replicated the primary analysis: (1) by changing the case definition of carditis within 28 days since the second dose into 14 days to ensure the causality assessment was stringent; and (2) by excluding 10% of samples with an extremely long interdose interval (e.g., dosing interval more than 190 days). The frequency of the interdose interval for adolescents is presented in Supplementary Fig. 1.

A detailed sample size calculation is attached in Supplementary Fig. 2. All analyses were performed in R version 4.1.0 (R Foundation for Statistical Computing, Vienna, Austria). Results were conducted independently by two researchers (M.F. and K.P.).

### Reporting summary

Further information on research design is available in the Nature Research Reporting Summary linked to this article.

### Supplementary information

REPORTING SUMMARY

Appdenxi tables and figure2

## Data Availability

Data used for this study will not be available to others as the data custodians (the Hospital Authority and the Department of Health of Hong Kong SAR) have not been permitted to share them due to patient confidentiality and privacy protection. Requests for data access could be submitted to the Central Panel on Administrative Assessment of External Data Requests of the Hospital Authority (hacpaaedr@ha.org.hk). As the data provided will be customized for the specific purpose of each project, the time duration required to process such requests may vary. Upon data request approval, no sharing of such data with third parties is allowed.
