# Supplementary material for: Risk of carditis among adolescents after extending the interdose intervals of BNT162b2
Source: NPJ Vaccines. 2024 Feb 14;9:31. doi: 10.1038/s41541-023-00789-6 (PMC10866879; doi:10.1038/s41541-023-00789-6)

# Supplementary materials

## Supplementary table 1 Recommended dose interval for priming doses in different countries

|  | **Vaccine types** | **Age group** | **Optimal / Recommended interval** | **Minimum / at least interval** |
| --- | --- | --- | --- | --- |
| **UK ^1^** | BNT162b2 bivalent or Monovalent XBB.1.5 BNT162b2 | 12-17 years | Three months | Three months |
| **US ^2^** | Monovalent XBB.1.5 BNT162b2 | ≥12 years | Get 1 update monovalent XBB.1.5 dose regardless of whether they’ve got COVID-19 vaccines before September 12, 2023 |  |
|  | Monovalent XBB.1.5 mRNA-1273 | ≥12 years |  |  |
| **Canada ^3^** | Monovalent XBB.1.5 BNT162b2 | ≥12 years | 8 weeks | N/A |
|  | BNT162b2 bivalent | ≥12 years | 8 weeks | 21 days |
|  | Monovalent XBB.1.5 mRNA-1273 | ≥12 years | 8 weeks | 28 days |
|  | mRNA-1273 bivalent | ≥12 years | 8 weeks | N/A |
| **Australia ^4^** | BNT162b2 bivalent | ≥12 years | 8 weeks | 21 days |
|  | mRNA-1273 bivalent | ≥12 years | 8 weeks | 28 days |
| **Norway ^5^** | BNT162b2 | 12-17 years | 8-12 weeks (should be vaccinated with one dose) | 21 days |
| **Finland ^6^** | BNT162b2 | 12-17 years | 6-12 weeks |  |
|  | mRNA-1273 | 12-17 years | 6-12 weeks (not offered to boys under the age of 30) |  |
|  | mRNA-1273 bivalent | 12-30 years | 4-6 weeks |  |
| **Singapore ^7^** | BNT162b2 | ≥5 years | 8 weeks | 21 days |
| **Taiwan ^8^** | BNT162b2 | 12-17 years | 12 weeks |  |
| **Japan ^9^** | Monovalent XBB.1.5 BNT162b2 | ≥12 years | 3 weeks |  |
| **HK ^10^** | BNT162b2 or BNT162b2 bivalent | 5-17 years | 8 weeks | 21 days |
| * The information in the table is up to date as 30th October 2023 when the manuscript is submitted for publication. | | | | |

**References**

1. Greenbook - chapter 14a - COVID-19 - SARS-CoV-2. *gov.uk* (Updated: 4th September 2023). Available at: <https://assets.publishing.service.gov.uk/government/uploads/system/uploads/attachment_data/file/1186479/Greenbook-chapter-14a-4September2023.pdf>. (Accessed: 30th October 2023)

2. Stay up to date with covid-19 vaccines. *Centers for Disease Control and Prevention* (Updated: 4th October 2023). Available at: https://www.cdc.gov/coronavirus/2019-ncov/vaccines/stay-up-to-date.html#footnote01. (Accessed: 30th October 2023)

3. COVID-19 vaccine: Canadian Immunization Guide. *Canada.ca* (Updated: 27th October 2023). Available at: https://www.canada.ca/en/public-health/services/publications/healthy-living/canadian-immunization-guide-part-4-active-vaccines/page-26-covid-19-vaccine.html#a5.2. (Accessed: 30th October 2023)

4. Australian Government Department of Health and Aged Care. Clinical recommendations for covid-19 vaccines. *Australian Government Department of Health and Aged Care* (Updated: 5th October 2023). Available at: https://www.health.gov.au/our-work/covid-19-vaccines/advice-for-providers/clinical-guidance/clinical-recommendations#primary-course-recommendations. (Accessed: 30th October 2023)

5. Coronavirus vaccine - information for the public. *Norwegian Institute of Public Health* (Updated: 18th September 2023). Available at: https://www.fhi.no/en/id/corona/coronavirus-immunisation-programme/coronavirus-vaccine/#vaccination-of-children-and-adolescents. (Accessed: 30th October 2023)

6. Coronavirus vaccinations for children and young people - THL. *Finnish Institute for Health and Welfare* (Updated: 3rd May 2023). Available at: https://thl.fi/en/web/infectious-diseases-and-vaccinations/what-s-new/coronavirus-covid-19-latest-updates/vaccines-and-coronavirus/coronavirus-vaccinations-for-children-and-young-people. (Accessed: 30th October 2023)

7. VACCINATION INFORMATION SHEET PFIZER-BIONTECH / COMIRNATY COVID-19 VACCINE. *Ministry of Health Singapore* (Updated: 28th October 2023). Available at: https://file.go.gov.sg/visp.pdf. (Accessed: 30th October 2023)

8. Pfizer-BioNTech COVID-19 Vaccine Information Sheet for Student Immunization (ages 5-17). *Taiwan Centers for Disease Control, Ministry of Health and Welfare* (Updated: 14th June 2022). Available at: https://www.cdc.gov.tw/Uploads/1fe80a5f-9765-43a2-bce7-cf430da10efc.pdf. (Accessed: 30th October 2023)

9. Information on primary vaccinations (1st and 2nd dose). *Ministry of Health, Labour and Welfare* (2023). Available at: https://www.mhlw.go.jp/stf/seisakunitsuite/bunya/0000121431_00218.html. (Accessed: 30th October 2023)

10. How many doses of COVID-19 vaccine are recommended for me? *Covid-19 vaccination programme* (Updated: 31th July 2023). Available at: https://www.chp.gov.hk/en/features/106951.html. (Accessed: 30th October 2023)

## Supplementary table 2 Incident carditis cases within 28 days following the second dose of BNT162b2

|  | **Male** | **Female** | **Total** |
| --- | --- | --- | --- |
| Age (ys, mean ± standard deviation) | 15.31±1.79 | 15.39±1.81 | 15.35±1.80 |
| Overall number of cases | 41 | 8 | 49 |
| Overval population of vaccinated adolecents | 71769 | 59201 | 130970 |
| Cumulative Incidence (per 100,000 persons with 95% CI) | 57.13 (41.00,77.50) | 13.51 (5.83,26.63) | 37.41 (27.68,49.46) |
| Days between first and second dose – 21-27 days | | | |
| Number of cases | 34 | 4 | 38 |
| Total number of vaccinated adolescents | 38531 | 32613 | 71144 |
| Cumulative Incidence (per 100,000 persons with 95% CI) | 88.24 (61.11,123.31) | 12.27 (3.34,31.4) | 53.41 (37.8,73.31) |
| Days between first and second dose – 28-55 days | | | |
| Number of cases | 0 | 0 | 0 |
| Total number of vaccinated adolescents | 2038 | 1820 | 3858 |
| Cumulative Incidence (per 100,000 persons with 95% CI) | - | - | - |
| Days between the first and second dose – 56 days above | | | |
| Number of cases | 7 | 4 | 11 |
| Total number of vaccinated adolescents | 31200 | 24768 | 55968 |
| Incidence (per 100,000 persons with 95% CI) | 22.44 (9.02,46.23) | 16.15 (4.4,41.35) | 19.65 (9.81,35.17) |
| Cumulative incidence with an exact 95% confidence interval (CI) was estimated based on Poisson distribution | | | |

## Supplementary table 3 ICD codes, Drug names, and British National Formulary (BNF) code used to identify disease and prescriptions

| **Description** | **Codes** | |
| --- | --- | --- |
|  | **ICD-9-CM codes** | |
| *Any infections* | 001-139 | |
| *Cardiovascular disease* |  |  |
| Vascular disease | 410, 411, 412, 413, 413, 443.8, 443.9 | |
| Myocardial infarction | 410 | |
| Peripheral vascular disease | 441, 443.9, 785.4, | |
| Coronary artery disease | 414.01 | |
| Arrhythmia | 427, 426, 794.3, 785.0 | |
|  | **British National Formulary** | |
| *Cardiovascular prescription* |  |  |
| Statin | 2.12 | |
| Angiotensin-converting enzyme | 2.5.5.1 | |
| Angiotensin Receptor Blockers | 2.5.5.2 | |
| Digoxin |  |  |
| Diuretics | 2.2 | |
| Anticoagulant | 2.8.2 | |
| Antiplatelet medications | 2.9 | |
| Beta-blocker | 2.4 | |
| Calcium channel blockers | 2.6.2 | |

## Supplementary figure 1 Frequency plot of interdose intervals between two priming doses of BNT162b2 in adolescents


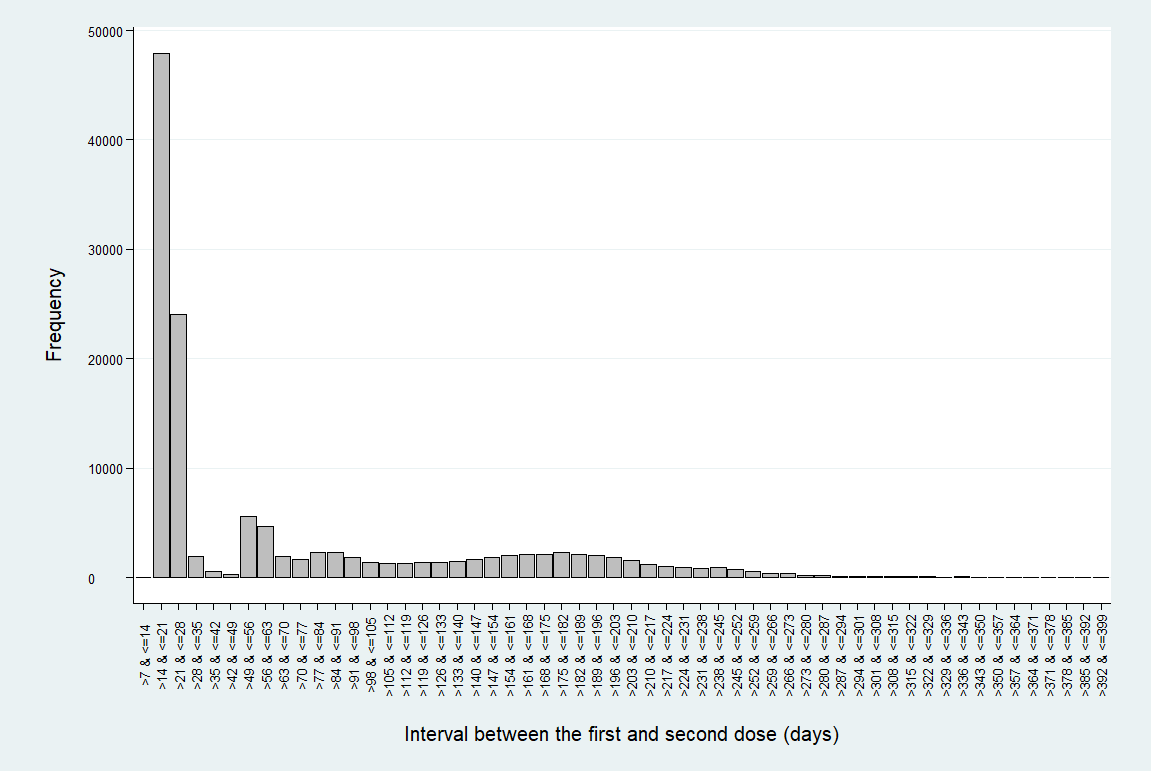


Note: The interquartile range of the interdose interval in Hong Kong for adolescents is 21-115 days

## ***Supplementary figure 2 Sample size calculation for the case-control design***

Our preliminary result showed that the unadjusted odds ratio for the risk of carditis after the second dose BNT162b2 was 0.33. Given that the prevalence of the extended dose was 44% of the Hong Kong population aged 12-17 years old, matched sets (1 case and 10 controls) ranging from 18 to 47 were required to achieve 60% to 95% power to detect the odds ratios of 0.33 at 0.05 significance level. With a power of 80%, the required sample sets are 28 (28 cases and 280 controls). The line indicates the required sample sets plotted for power from 0.50 to 0.95 by 0.05. The sample size calculation was conducted using the R package “powerSurvEpi”.


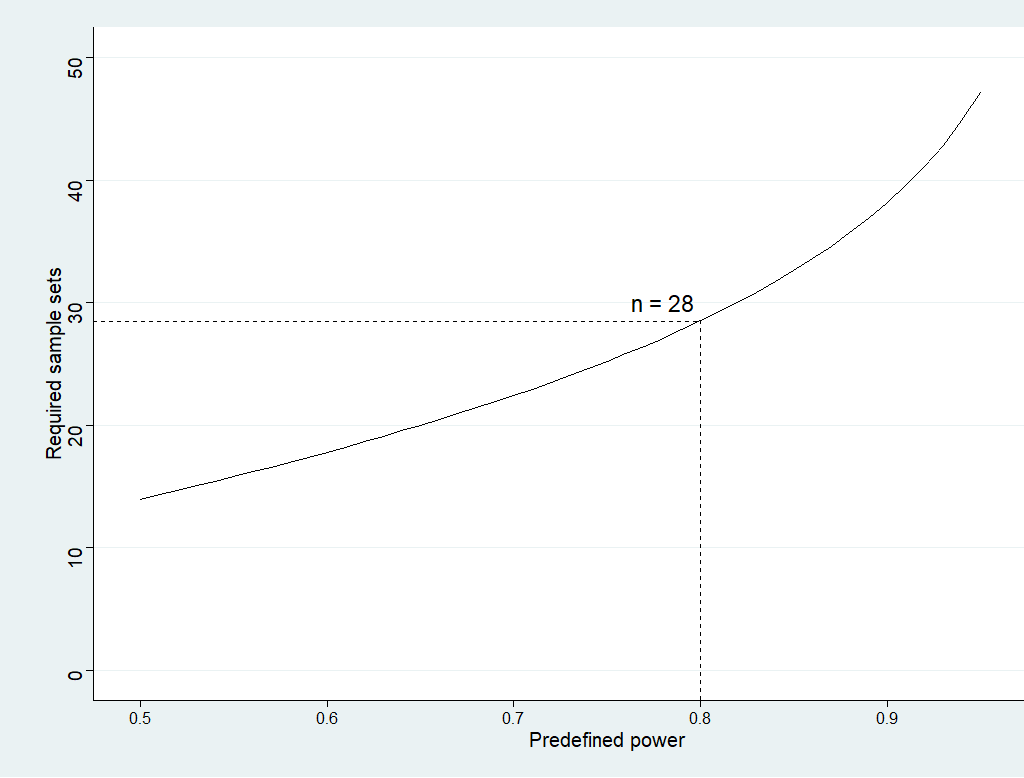

Supplement: Supplementary file 2 — Appdenxi tables and figure2 [file 41541_2023_789_MOESM2_ESM.docx]
